# The association between heart rate behavior and gait performance: The moderating effect of frailty

**DOI:** 10.1371/journal.pone.0264013

**Published:** 2022-02-16

**Authors:** Kayleigh Ruberto, Hossein Ehsani, Saman Parvaneh, Jane Mohler, Mindy Fain, Nancy K. Sweitzer, Nima Toosizadeh

**Affiliations:** 1 Department of Biomedical Engineering, University of Arizona, Tucson, Arizona, United States of America; 2 Kinesiology Department, University of Maryland, College Park, MD, United States of America; 3 Edwards Life Sciences, Irvine, CA, United States of America; 4 Division of Geriatrics, General Internal Medicine and Palliative Medicine, Department of Medicine, University of Arizona, Tucson, Arizona, United States of America; 5 Arizona Center on Aging, Department of Medicine, University of Arizona, Tucson, Arizona, United States of America; 6 Arizona Sarver Heart Center, Department of Medicine, University of Arizona, Tucson, Arizona, United States of America; University of Groningen: Rijksuniversiteit Groningen, NETHERLANDS

## Abstract

**Introduction:**

Research suggests that frailty not only influence individual systems, but also it affects the interconnection between them. However, no study exists to show how the interplay between cardiovascular and motor performance is compromised with frailty.

**Aim:**

To investigate the effect of frailty on the association between heart rate (HR) dynamics and gait performance.

**Methods:**

Eighty-five older adults (≥65 years and able to walk 9.14 meters) were recruited (October 2016—March 2018) and categorized into 26 non-frail (age = 78.65±7.46 years) and 59 pre-frail/frail individuals (age = 81.01±8.17) based on the Fried frailty phenotype. Participants performed gait tasks while equipped with a wearable electrocardiogram (ECG) sensor attached to the chest, as well as wearable gyroscopes for gait assessment. HR dynamic parameters were extracted, including time to peak HR and percentage increase in HR in response to walking. Using the gyroscope sensors gait parameters were recorded including stride length, stride velocity, mean swing velocity, and double support.

**Results:**

Among the pre-frail/frail group, time to peak HR was significantly correlated with all gait parameters (*p*<0.0001, *r* = 0.51–0.59); however, for the non-frail group, none of the correlations between HR dynamics and gait performance parameters were significant (*p*>0.45, *r* = 0.03–0.15). The moderation analysis of time to peak HR, demonstrated a significant interaction effect of HR dynamics and frailty status on walking velocity (*p*<0.01), and the interaction effect was marginally non-significant for other gait parameters (*p*>0.10).

**Conclusions:**

Current findings, for the first time, suggest that a compromised motor and cardiac autonomic interaction exist among pre-frail/frail older adults; an impaired HR performance (i.e., slower increase of HR in response to stressors) may lead to a slower walking performance. Assessing physical performance and its corresponding HR behavior should be studied as a tool for frailty screening and providing insights about the underlying cardiovascular-related mechanism leading to physical frailty.

## Introduction

Frailty is a geriatric syndrome related to diminished physiological reserves and is defined as the presence of weakness, slowness, exhaustion, low physical activity, and unintentional weight loss by Fried et al. [1]. Frailty is shown to put older adults at higher risk for adverse health outcomes such as higher rates of hospitalization and readmission, adverse treatment outcomes, longer hospital stays, and increased mortality [1, 2]. Lack of physiological reserves makes frail individuals less likely to withstand stressors [3], and there are a large range of physical and physiological markers to measure the lack of reserve among older adults [4, 5]. Specifically, among physical markers, parameters related to lower-extremity muscular performance are strongly associated with frailty, such as gait speed [6, 7]. It is now well established that muscle loss and weakness (sarcopenia and dynapenia) are the main symptoms of frailty, caused by inflammatory (elevated interleukin 6 (IL-6), C-reactive protein (CRP), and tumor necrosis factor alpha (TNFα)), metabolic (deficiencies of various mitochondrial subunits), and hormonal derangements (cortisol and testosterone) that shift homeostasis from an anabolic to a catabolic state [8–16]. On the other hand, similar muscle dysfunction has been observed in cardiac frailty with the same inflammatory, metabolic, and hormonal contributors, further exacerbated by the lack of cardiovascular reserve and a compromised autonomic nervous system (ANS) [17–23]. Accordingly, heart rate (HR) response to stressors may also be an underlying reason for the deterioration of physical performance such as walking.

HR measures have been previously demonstrated to be associated with frailty in older adults [24, 25]. Specifically, it was found that complexity of HR, heart rate variability (HRV), and changes in HRV while performing physical activity (HR dynamics: the amount of increase and decrease in HR and the time to take to maximum and minimum HR during activity and recovery) were reduced in pre-frail and frail individuals, compared to non-frails, which suggests impairments in autonomic nervous system performance due to frailty [2, 25]. In addition, frail older adults are more likely to develop cardiovascular diseases such as myocardial infarction and heart diseases due to malfunctions in the sinoatrial node caused by changes in action potential morphology and the electrical conduction pathway [26–28]. Further, recent work suggested changes in interconnection between motor and autonomic cardiac performances with aging [29]. In the LINK-HF study cohort, wearable sensors on the chest were utilized to collect HR and activity (e.g., walking) in combination with machine learning approaches to predict risk of re-hospitalization in older adults with heart failure [30, 31]. More recently, a 6-minute walk test app has been validated for the Apple Watch that incorporates both motor and HR behaviors for use in patients with cardiovascular disease [32]. Nevertheless, the association between HR measures and deterioration in physical activity, such as gait performance, across frailty groups is not clear.

The goal of the current study was to determine the association between gait performance and HR response to walking and determine if this association is dependent on the frailty level. We have previously demonstrated strong associations between HR dynamics with frailty [2]. Our previous findings suggest compromised HR dynamics, which was quantified by a slower and smaller change in HR during and after walking. The first hypothesis of the current study was that there would be a significant interaction effect of frailty and HR dynamics on walking performance. As a secondary hypothesis, it is expected that HR dynamics are significantly correlated with gait performance among pre-frail and frail older adults, while this relationship is weaker among non-frail individuals. If these hypotheses are confirmed, they would suggest that each of the HR and motor performance measures provides a distinctive measure of frailty, which can ultimately be used together for enhancing frailty identification among older adults.

## Methods

### Study design and participants

This was a cross-sectional observational study. Participants were recruited between October 2016 and March 2018, from the Arizona Frailty and Fall Cohort. Sources of recruitment included primary, secondary, and tertiary health care settings, community providers, assisted living facilities, retirement homes, and aging service organizations in Tucson, Arizona, United States. Participants were contacted via flyers, emails, and phone calls. Inclusion criteria were: 1) being 65 years or older; and 2) the ability to walk a minimum distance of 9.14 meters (30 feet) with or without an assistive device. Exclusion criteria were: 1) gait or severe motor function disorders (e.g., Parkinson’s disease, Multiple Sclerosis, or recent stroke); 2) cognitive impairment identified by a Mini-Mental State Examination (MMSE) score ≤ 23; 3) usage of *β*-blockers or similar medications that can influence HR; 4) diseases/disorders that can directly influence HR (including arrhythmia and use of pacemaker); and 5) terminal illness. Before participating, subjects were informed of the study protocol and consenting process, and they were given time to read the consent and ask questions before participation. The study was approved by the University of Arizona Institutional Review Board and written informed consent according to the principles expressed in the Declaration of Helsinki [33] was obtained from eligible subjects before participation; a signed copy of the consent was provided to each participant. Data collection was performed at participants’ homes by two research staff who were blinded of the frailty status of participants.

### Clinical measures and frailty assessment

Clinical measures collected included: 1) MMSE and Montreal Cognitive Assessment (MoCA) [34, 35]; 2) comorbidity based on Charlson Comorbidity Score (CCI) [36]; 3) depression using Patient Health Questionnaire (PHQ-9) [37]; 4) number of falls and fall risk based on Stopping Elderly Accidents, Death & Injury (STEADI) [38, 39]; and 5) Falls Efficacy Scale-International (FES-I) [40]. These measures were collected because they could potentially influence physical activity and the cardiovascular system performance, and accordingly were considered as adjusting variables in the moderation analysis.

Frailty was defined according to previously validated criteria [1], which determines frailty level based on: 1) self-reported unintentional weight loss of 4.54 kilograms (10 pounds) or more within the prior year; 2) weakness based on a grip strength test; 3) slow walking speed; 4) self-reported exhaustion based on a short two-question version of Center for Epidemiological Studies Depression (CES-D) scale; and 5) low energy expenditure based on the short version of Minnesota Leisure Time Activity questionnaire [41]. Participants that did not meet any of the criteria were categorized as non-frail, pre-frail if they met one or two criteria, and frail if they met three or more criteria.

### Gait tests and HR measures

Participants walked 4.57 meters (15 feet) in their home at a self-selected normal pace, as recommended in previous work [1]. Participants wore two motion sensors, one on each shin above the ankle, including a triaxial accelerometer and gyroscope (LEGSys^TM^, BioSensics, Boston, MA) [42, 43]. Motion sensors were used to record gait parameters, including: stride velocity normalized with height, mean swing velocity during the swing phase, stride length normalized with height, and double support duration as a percentage of the gait cycle, based on previously validated algorithms (Table 1) [42, 44, 45]. The mean of all these parameters were calculated for the whole duration of walking.

**Table 1:** Definition of gait and heart rate (HR) parameters.

**Gait parameters**	
Stride velocity	Mean value of distance covered by each stride over time (normalized with height)
Mean swing velocity	Mean value of shin angular velocity peaks during swing phase
Stride length	Mean value of distance traveled by the same foot between two successive heel contacts (normalized with height)
Double support duration	Mean value of duration of initial and terminal double support (both feet in contact with the ground) as a percentage of the gait cycle time
**HR dynamic parameters**	
Time to peak HR	Elapsed time to reach maximum HR during task with reference to minimum baseline HR
Percent increase	Increase in HR during the task compared to minimum baseline HR as the percentage of minimum baseline HR
Increase rate	Rate of increase in HR over time
**Baseline HR parameters**	
HR mean	Mean value of number of heart beats per minute
RR mean	Mean of beat-to-beat RR interval
RR CV	Coefficient of variation (CV: standard deviation divided by mean) of RR interval
RMSSD	Root mean square of successive heartbeat interval differences

HR: Heart rate.

CV: Coefficient of variance.

SD: Standard deviation.

RMSSD: Root mean square of successive differences.

**Fig 1:**
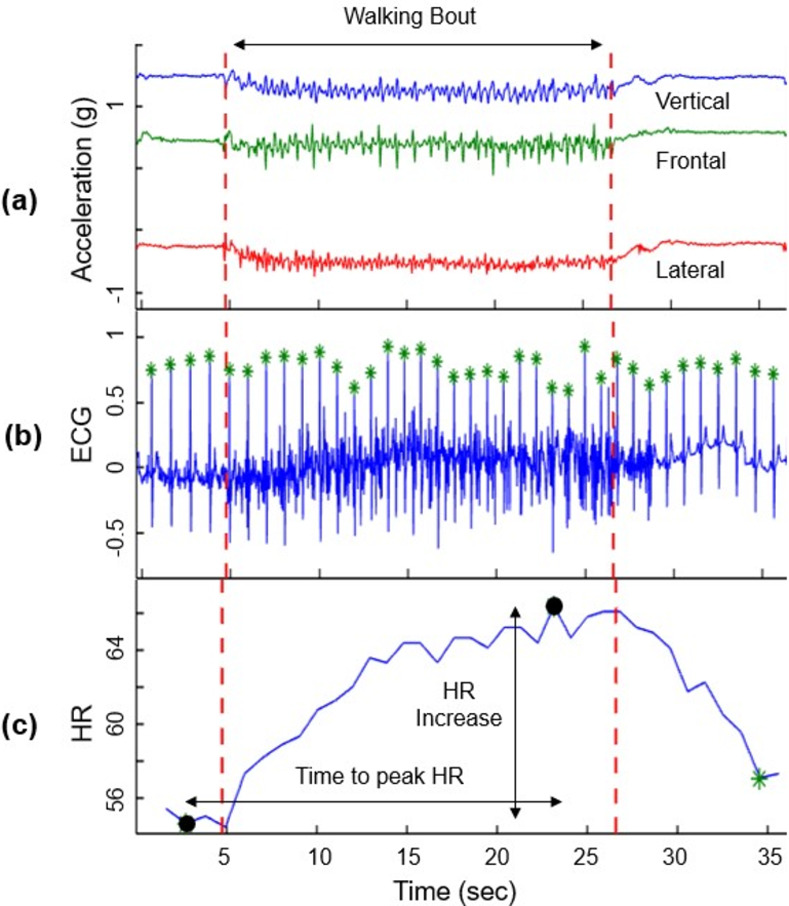
MATLAB output. Graph A: acceleration versus time graph to determine the period of walking; Graph B: ECG time-series, from which, QRS detection process was performed to extract HR time series; and Graph C: HR time series and HR dynamic parameters in response to the walking task.

While walking, participants wore an electrocardiogram (ECG)/accelerometer sensor to assess their HR. One channel ECG was recorded using two electrodes; one electrode was placed medial-supraclavicularly on the left side of the torso and the other one on the left side, laterally under the rib cage. To extract the ECG outcomes, the synchronized embedded accelerometer data was used to extract a five second baseline before the task and the exact starting and ending time of the walking task. The ECG time series was used to identify the QRS peaks, representing ventricular depolarization, which then produced the RR interval and HR time series (Fig 1). Two types of outcomes were extracted from the ECG data, including: 1) baseline HR parameters; and 2) HR dynamics (HR response to the walking activity). As presented in Table 1, the HR dynamic parameters represent the change in HR due to walking including: time to peak HR, percent increase in HR due to walking with respect to five-second baseline measure of HR, and HR increase rate (i.e., HR increase divided by time to peak HR). The baseline HR parameters included the mean HR and RR, RR coefficient of variation (CV), and root-mean-square of successive differences (RMSSD) of the RR interval.

### Statistical analysis

Analysis of variance (ANOVA) models were used to evaluate the differences in all continuous parameters of demographic, clinical measures, and gait and HR parameters between two frailty groups. Chi-square (χ^2^) tests were used to assess differences in sex and fall risk categories among frailty groups. To assess the association between gait performance measures and HR parameters, first, Pearson correlation tests were explored between each HR and gait performance parameters, for all participants and within each frailty group. In this step, HR parameters with significant association with gait performance measures were selected for the moderation analysis.

A moderation analysis was performed to assess how frailty status can influence the association between HR and gait performance. For this, frailty status was considered a moderator variable and the interaction effects of frailty and HR parameters on gait measures were calculated [46, 47]. Four separate moderation analyses were performed considering each of the four gait parameters as the dependent variable, including stride velocity, mean swing velocity, stride length, and double support duration. Within each analysis, three models were developed within three steps. In Step 1, the selected HR parameters from the Pearson correlation analysis along with demographics and clinical measures with significant association with frailty were considered as independent variables in a multivariable ANOVA model; each of the gait parameters was considered as the dependent variable. HR parameters with significant association with gait parameters within the multivariable ANOVA models were selected as candidates for the next step. Then, HR and demographic parameters were selected using a stepwise method based on Akaike information criterion (AIC) values. Of note, due to relatively small sample size, to avoid over-fitting, only one HR parameter was included in each stepwise model. In Step 2, frailty status was added to the list of independent variables from Step 1. Finally, in Step 3, the HR parameter and frailty interaction effect was added as an additional independent variable to the model from Step 2. Goodness of fit was assessed for each model using residual plot. Root-mean-squared-error (RMSE), *R*^2^, and AIC were reported for each step. Statistical analyses were done using JMP (Version 15, SAS Institute Inc., Cary, NC, USA), and statistical significance was concluded when *p*<0.05.

## Results

### Participants

Eighty-five participants were recruited, including 26 non-frail (age = 77.65±7.32 years), 52 pre-frail (age = 80.25±8.23 years), and seven frail (age = 86.71±3.61 years). Due to the small sample of frail participants, frailty status was dichotomized into non-frail and pre-frail/frail groups. Comorbidity, depression, FES-I, and number of falls were significantly different between frailty groups (*p*<0.01, Table 2), all other demographics were not significantly different between groups (*p*>0.12, Table 2).

**Table 2:** Participant demographic, clinical measures, and gait and HR parameters.

Demographic Information	Non-frail (n = 26)	Pre-frail/Frail (n = 59)	*p*-value
Male (% of group)	9 (35%)	16 (27%)	0.49
Age, years (SD)	78.65 (7.46)	81.01 (8.17)	0.21
Stature, cm (SD)	167.15 (9.97)	163.74 (10.01)	0.15
Body mass, kg (SD)	71.51 (13.99)	74.75 (19.20)	0.44
BMI, kg/m^2^ (SD)	25.73 (5.68)	27.67 (5.93)	0.16
**Clinical Measures**			
MMSE score, 0–30 (SD)	29.50 (0.71)	29.10 (1.30)	0.15
MoCA score, 0–30 (SD)	26.04 (2.95)	24.98 (2.86)	0.12
CCI score, 0–29 (SD)	1.81 (1.92)	3.64 (2.66)	<0.01*
PHQ-9 score, 0–27 (SD)	1.04 (1.06)	2.97 (3.65)	0.01*
STEADI categories			0.09
Low, n	17	28	
Moderate, n	6	16	
High, n	3	15	
FES-I score, 7–28 (SD)	9.07 (2.10)	11.85 (5.26)	0.01*
Number of falls, n (SD)	0.46 (0.81)	1.10 (1.45)	0.04*
**Gait parameters**			
Stride velocity, 1/second (SD)	0.48 (0.14)	0.38 (0.13)	<0.01* (0.74)
Mean swing velocity, deg/second (SD)	38.49 (3.32)	36.13 (3.53)	<0.01* (0.69)
Stride length, % height (SD)	56.62 (16.10)	35.75 (12.35)	<0.01* (0.70)
Double support duration, % (SD)	23.01 (6.65)	27.73 (7.07)	<0.01* (0.68)
**HR dynamic parameters**			
Time to peak HR, second (SD)	6.03 (2.34)	8.96 (3.70)	<0.01* (0.93)
Percent increase, % (SD)	22.03 (19.36)	11.99 (8.67)	<0.01* (0.67)
Increase rate, beats per minute/second (SD)	3.78 (5.75)	1.25 (1.20)	<0.01* (0.61)
**Baseline HR parameters**			
HR mean, beats per minute (SD)	75.08 (11.98)	78.67 (15.41)	0.25 (0.26)
RR mean, second (SD)	0.82 (0.12)	0.79 (0.15)	0.30 (0.22)
RR CV, % (SD)	4.21 (5.56)	1.94 (2.15)	<0.01* (0.54)
RMSSD, millisecond (SD)	43.43 (61.47)	21.12 (29.25)	0.01* (0.46)

SD: standard deviation.

BMI: Body Mass Index.

MMSE: Mini-Mental State Examination (score from 0 for cognitively impaired to 30 for cognitively normal with a cutoff of 24).

MoCA: Montreal Cognitive Assessment (score from 0 for cognitively impaired to 30 for cognitively normal with a cutoff of 26).

CCI: Charlson Comorbidity Index (representing the number of comorbidity).

PHQ-9: Patient Health Questionnaire (score from 0 for depression to 27 for healthy mental condition).

STEADI: Stopping Elderly Accidents, Deaths & Injury.

FES-I: Falls Efficacy Scale-International (score from 7 for no fear of falling to 28 for extreme level of fear of falling).

HR: Heart rate.

CV: Coefficient of variance.

SD: Standard deviation.

RMS: Root mean square.

A significant difference between groups is denoted by the asterisk symbol.

### Correlation between gait and HR parameters

**Fig 2:**
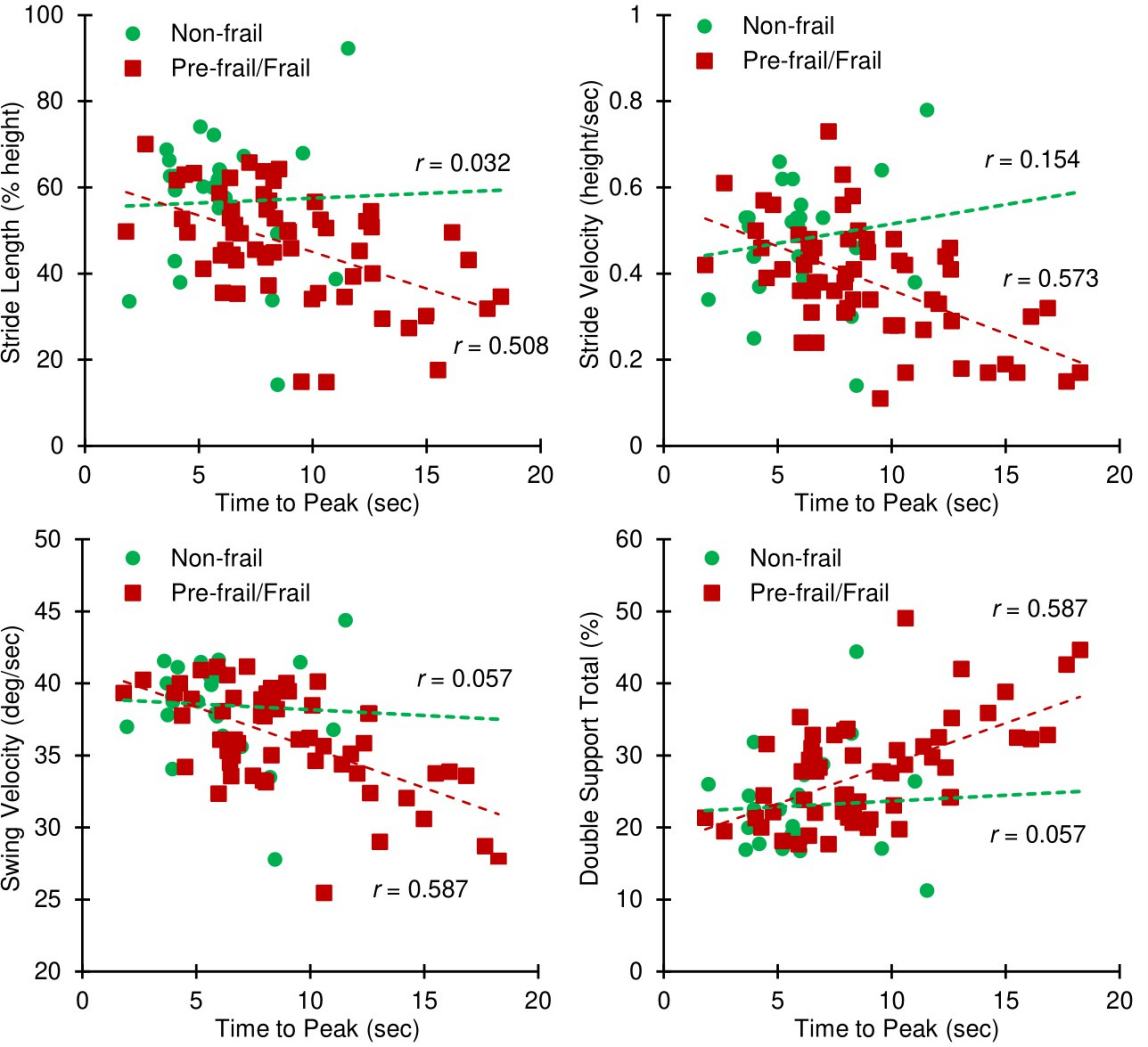
Correlation analyses. Correlation between gait parameters and HR response to walking (time to peak HR) among non-frail and pre-frail/frail groups.

All HR and gait parameters are reported in Table 2. Among all non-frail and pre-frail/frail participants, time to peak HR was significantly correlated with all gait parameters of stride length, stride velocity, mean swing velocity, and double support duration (*p*<0.0001, *r* = 0.43–0.53, Table 3). All these correlations were significant for the pre-frail/frail group only (*p*<0.0001, *r* = 0.51–0.59, Fig 2). Similar results were observed between HR increase rate with mean swing velocity and double support duration for all participants and the pre-frail/frail group (Table 3). On the other hand, none of the correlations were significant for the non-frail group for any of these HR response parameters (*p*>0.45, *r* = 0.03–0.15, Table 3). Further, none of the baseline HR parameters were significantly associated with gait performance, neither for all participants (*p*>0.18, *r*<0.07), nor for each of the frailty group separately (*p*>0.41, *r*<0.11, S1 Table). Time to peak HR was selected for the moderation analysis, since it showed significant association with all gait parameters when adjusted with demographic information and clinical measures (*p*<0.0001).

**Table 3:** Correlation analyses between HR response (time to peak HR and increase rate) and gait parameters.

	All Participants *r-*value *(p-*value)		Non-frail *r-*value *(p-*value)		Pre-frail/frail *r-*value *(p-*value)	
	Time to Peak HR	Increase Rate	Time to Peak HR	Increase Rate	Time to Peak HR	Increase Rate
Stride Length	0.4268 (<0.001*)	0.1481 (0.1841)	0.0322 (0.8758)	0.0788 (0.7143)	0.5079 (<0.001*)	0.0080 (0.9527)
Stride Velocity	0.4754 (<0.0001*)	0.1549 (0.1648)	0.1539 (0.4528)	0.1058 (0.6226)	0.5733 (<0.0001*)	0.1534 (0.2502)
Mean Swing Velocity	0.5314 (<0.0001*)	0.2786 (0.0113*)	0.0568 (0.7829)	0.0677 (0.7530)	0.5868 (<0.0001*)	0.2908 (0.0268*)
Double Support Duration	0.5314 (<0.0001*)	0.2786 (0.0113*)	0.0569 (0.7824)	0.0679 (0.7526)	0.5868 (<0.0001*)	0.2908 (0.0268*)

Significant values are denoted by the asterisk symbol. See the S1 Table for correlation results between baseline HR and gait parameters.

### Moderation analysis

Results of the moderation analysis showed that time to peak HR was significantly associated to all gait performance parameters within multivariable models (Step 1), as well as multivariate models in combination with frailty as an additional independent variable (Step 2, *p*<0.0012, Table 4). When the frailty and time to peak HR interaction effect added as the third independent variable (Step 3), independent association between time to peak HR and gait parameters remained significant only for mean swing velocity and double support duration (*p*<0.03, Table 4). Further, significant frailty and time to peak HR interaction effect was observed in association with the walking stride velocity (*p*<0.01).

**Table 4:** Moderation analyses for HR dynamics (time to peak HR).

	Parameter Estimates (*p*-value)	*R* ^2^	RMSE *	AIC
Stride Velocity (height/s)				
Step 1				
Time to Peak	<0.0001*	0.2250	0.1234	-110.15
Step 2				
Time to Peak	0.0001*	0.2492	0.1223	-110.54
Frailty	0.1153			
Step 3				
Time to Peak	0.3023	0.3106	0.1179	-115.53
Frailty	0.0067*			
Time to Peak*Frailty	0.0088*			
Mean Swing Velocity				
Step 1				
Time to Peak	<0.0001*	0.2824	3.0862	437.08
Step 2				
Time to Peak	<0.0001*	0.2943	3.791	437.85
Frailty	0.2421			
Step 3				
Time to Peak	0.0255*	0.3187	3.0440	437.12
Frailty	0.0626			
Time to Peak*Frailty	0.0924			
Stride Length (% height)				
Step 1				
Time to Peak	<0.0001*	0.1822	12.9743	681.20
Step 2				
Time to Peak	0.0012*	0.2117	12.8149	680.27
Frailty	0.0831			
Step 3				
Time to Peak	0.2133	0.2368	12.6868	679.78
Frailty	0.0210*			
Time to Peak*Frailty	0.1065			
Double Support Duration				
Step 1				
Time to Peak	<0.0001*	0.2824	6.1722	554.90
Step 2				
Time to Peak	<0.0001*	0.2943	6.1578	555.68
Frailty	0.2424			
Step 3				
Time to Peak	0.0255*	0.3187	6.0878	554.95
Frailty	0.0627			
Time to Peak*Frailty	0.0926			

* RMSE: root mean square error, with units of 1/second, deg/second, % height, and % for stride velocity, mean swing velocity, stride length, and double support duration, respectively.

AIC: Akaike information criterion.

A significant difference is denoted by the asterisk symbol.

## Discussion

### Interplay between frailty, HR, and gait performance

As hypothesized, we observed a significant effect of frailty level on the association between HR dynamics and walking performance (interaction effect of frailty and HR dynamics on gait performance), showing that the association between gait performance and HR behavior was stronger in pre-frail/frail than in non-frail older adults. One explanation for this observation may be the fact that the short-duration walking task was not intense enough for non-frail older adults to reach their limits of HR performance. On the other hand, among pre-frail/frail older adults, we observed inability to quickly increase the HR, which was associated with an impaired gait performance. Frailty is defined as a lack of physiological reserves [1], and its symptoms may not be limited to one specific system; as current findings suggested, frailty influence both cardiovascular system and the motor function. Nevertheless, no causal relationship between HR dynamics and gait performance can be concluded; it is not clear from the current findings whether the lack of cardiovascular reserves led to impairment in gait performance.

Several physiological mechanisms can be influenced by frailty. Considering the motor function system independently, previous studies have confirmed deterioration in gait performance with frailty, which was observed as gait spatial-temporal differences between non-frail, pre-frail, and frail individuals, including gait velocity, stride length, and double-support [48–50]. These studies reported on average 36% and 14% less gait velocity and stride length and 16% larger double-support among pre-frail/frail older adults compared to non-frail individuals [48–50], which are comparable to the current results (Table 2). These changes in gait performance partially due to dynapenia (i.e., loss of muscle strength and power) in pre-frail and frail older adults, which can lead to weakness, fatigue, and incapability of doing simple physical tasks such as walking [51, 52]. Several underlying factors can cause dynapenia in frailty, including inflammation and nutritional deficiencies [52], as well as changes in neuromuscular morphology and function (e.g., loss in the volume of motor units and the mass of type 2 fibers) [53, 54].

On the other hand, aging and frailty are associated with impaired performance of the cardiovascular system [55, 56]. Specifically, frailty can affect the autonomic nervous system performance and, consequently HR response [2, 25]. This has been observed in our previous research, where pre-frail/frail older adults showed a weaker and slower HR response to physical activity, compared to non-frail individuals (46% less increase in heart rate, and 49% slower occurrence of heart rate peak among pre-frail/frail when compared to non-frail older adults) [2]. Moreover, it has been observed that frailty can lead to an impaired orthostatic HR, which can consequently cause the systolic blood pressure to recover more slowly during an active stand [24]. The observed lack of heart performance due to frailty may be partially attributable to deficits in electrical conduction and action potential morphology [57], dynapenia that can affect cardiac muscle [58], and inflammatory cytokines [58].

As the above previous evidence demonstrated, several underlying mutual factors related to frailty can simultaneously affect both cardiovascular and motor systems. This observation confirmed the multi-dimensional characteristic of frailty effects on physiological systems, which was confirmed by current findings. In contrary to what was observed here for the association between time to peak HR and gait performance among frailty groups, Weiss et al. reported no interaction effect of frailty and cardiac function on exercise capacity within a seated step test [59]. In this study cardiac function was measured based on the chronotropic index, which is the index of HR reserve during the exercise session. We believe that the main reason for the observed discrepancy is related to the selection of HR parameters (i.e., HR response to activity); although, within the current study, time to peak response showed significant main and interaction effect (across frailty groups) with gait performance, none of the baseline HR parameters, even within our sample of data, showed significant association with gait performance (S1 Table). Although previous studies showed that frail older adults have higher HR and lower HRV during inactive baseline conditions [2, 24, 57, 59], the current findings suggest that these parameters may not be directly associated with physical activity impairments. In this regard, the HR response measures showed more promise for associating frailty with physical function impairment; nevertheless, this hypothesis needs to be further confirmed in future research.

### Limitations and future direction

Other than HRV, other measures of HR complexity during resting may provide meaningful assessment of autonomic system performance that are not calculated here [60]. However, longer than a five-second resting period is required to obtain reliable complexity measures such as sample or multiscale entropy. The frailty and HR complexity interaction on physical function should be investigated in future research. Also, although we validated HR dynamics outcomes for frailty assessment, their test-retest reliability should be investigated in future research. Further, due to the small number of frail participants pre-frail and frail groups were merged, and therefore, the trajectory of the interaction effect of frailty across non-frail, pre-frail, and frail individuals requires more investigations in future. Lastly, as a common step in HR monitoring, we manually inspected QRS detection for ECG data, since, inherently, noise due to motion artifacts exists in the data during physical activities such as walking task. Overall, 3% of the total data (three out of a total of 88 participants) were removed because the peak detection process could not be completed due to the noise problem.

## Conclusion and implications

In this study, we demonstrated a significant association between HR dynamic measures and walking, specifically time to peak HR, and gait performance among pre-frail/frail older adults. On the other hand, no significant association was observed between HR measures and gait performance among non-frail participants. This suggests a significant interaction effect of frailty on the performance of cardiovascular and motor systems. Our findings suggest that HR dynamic measures may provide a distinct measure of frailty that in combination with physical function assessments can potentially provide a practical multidimensional tool for assessing frailty. One advantage of the current approach was being objective, implementing sensor-based measurements instead of subjective questionnaires.

## Supporting information

S1 Data (XLSX)

S1 Table Correlation analyses between baseline HR and HR dynamic parameters with gait parameters for all participants and for each frailty group. Significant values are denoted by the asterisk symbol.
(DOCX)

## References

[1] Fried LP, Tangen CM, Walston J, Newman AB, Hirsch C, et al. (2001) Frailty in older adults: evidence for a phenotype. The Journals of Gerontology Series A: Biological Sciences and Medical Sciences 56: M146–M157. doi: 10.1093/gerona/56.3.m146 11253156

[2] Toosizadeh N, Ehsani H, Parthasarathy S, Carpenter B, Ruberto K, et al. (2021) Frailty and heart response to physical activity. Archives of gerontology and geriatrics 93: 104323. doi: 10.1016/j.archger.2020.104323 33340830

[3] Clegg A, Young J, Iliffe S, Rikkert MO, Rockwood K (2013) Frailty in elderly people. The lancet 381: 752–762. doi: 10.1016/S0140-6736(12)62167-9 23395245 PMC4098658

[4] Cohen HJ (2000) In search of the underlying mechanisms of frailty. The Journals of Gerontology Series A: Biological Sciences and Medical Sciences 55: M706–M708. doi: 10.1093/gerona/55.12.m706 11129391

[5] Borges L, Menezes R (2011) Definitions and markers of frailty: a systematic review of literature. Reviews in Clinical Gerontology 21: 67–77.

[6] Schwenk M, Howe C, Saleh A, Mohler J, Grewal G, et al. (2014) Frailty and technology: a systematic review of gait analysis in those with frailty. Gerontology 60: 79–89. doi: 10.1159/000354211 23949441 PMC4017858

[7] Thiede R, Toosizadeh N, Mills JL, Zaky M, Mohler J, et al. (2016) Gait and balance assessments as early indicators of frailty in patients with known peripheral artery disease. Clinical biomechanics 32: 1–7. doi: 10.1016/j.clinbiomech.2015.12.002 26775227 PMC4779419

[8] Yao X, Li H, Leng SX (2011) Inflammation and immune system alterations in frailty. Clinics in geriatric medicine 27: 79–87. doi: 10.1016/j.cger.2010.08.002 21093724 PMC3011971

[9] Hubbard RE, O’Mahony MS, Savva GM, Calver BL, Woodhouse KW (2009) Inflammation and frailty measures in older people. Journal of cellular and molecular medicine 13: 3103–3109. doi: 10.1111/j.1582-4934.2009.00733.x 19438806 PMC4516469

[10] Serviddio G, Romano AD, Greco A, Rollo T, Bellanti F, et al. (2009) Frailty syndrome is associated with altered circulating redox balance and increased markers of oxidative stress. International Journal of Immunopathology and Pharmacology 22: 819–827. doi: 10.1177/039463200902200328 19822098

[11] Schaap LA, Pluijm SM, Deeg DJ, Visser M (2006) Inflammatory markers and loss of muscle mass (sarcopenia) and strength. The American journal of medicine 119: 526. e529-526. e517. 10.1016/j.amjmed.2005.10.049 16750969

[12] Visser M, Pahor M, Taaffe DR, Goodpaster BH, Simonsick EM, et al. (2002) Relationship of interleukin-6 and tumor necrosis factor-α with muscle mass and muscle strength in elderly men and women: the Health ABC Study. The Journals of Gerontology Series A: Biological Sciences and Medical Sciences 57: M326–M332. doi: 10.1093/gerona/57.5.m326 11983728

[13] Cesari M, Penninx BW, Pahor M, Lauretani F, Corsi AM, et al. (2004) Inflammatory markers and physical performance in older persons: the InCHIANTI study. The Journals of Gerontology Series A: Biological Sciences and Medical Sciences 59: M242–M248. doi: 10.1093/gerona/59.3.m242 15031308

[14] Johar H, Emeny RT, Bidlingmaier M, Reincke M, Thorand B, et al. (2014) Blunted diurnal cortisol pattern is associated with frailty: a cross-sectional study of 745 participants aged 65 to 90 years. The Journal of Clinical Endocrinology & Metabolism 99: E464–E468. doi: 10.1210/jc.2013-3079 24564322

[15] O’Connell M, Tajar A, Roberts SA, Wu F (2011) Do androgens play any role in the physical frailty of ageing men? International journal of andrology 34: 195–211. doi: 10.1111/j.1365-2605.2010.01093.x 20722765

[16] López-Armada MJ, Riveiro-Naveira RR, Vaamonde-García C, Valcárcel-Ares MN (2013) Mitochondrial dysfunction and the inflammatory response. Mitochondrion 13: 106–118. doi: 10.1016/j.mito.2013.01.003 23333405

[17] Goldwater DS, Pinney SP (2015) Frailty in advanced heart failure: a consequence of aging or a separate entity? Clinical Medicine Insights: Cardiology 9: CMC. S19698. doi: 10.4137/CMC.S19698 26244037 PMC4501712

[18] Anker S, Ponikowski P, Clark AL, Leyva F, Rauchhaus M, et al. (1999) Cytokines and neurohormones relating to body composition alterations in the wasting syndrome of chronic heart failure. European heart journal 20: 683–693. doi: 10.1053/euhj.1998.1446 10208789

[19] Levine B, Kalman J, Mayer L, Fillit HM, Packer M (1990) Elevated circulating levels of tumor necrosis factor in severe chronic heart failure. New England Journal of Medicine 323: 236–241. doi: 10.1056/NEJM199007263230405 2195340

[20] McMurray J, Abdullah I, Dargie HJ, Shapiro D (1991) Increased concentrations of tumour necrosis factor in" cachectic" patients with severe chronic heart failure. Heart 66: 356–358. doi: 10.1136/hrt.66.5.356 1747295 PMC1024773

[21] Anker SD, Clark AL, Kemp M, Salsbury C, Teixeira MM, et al. (1997) Tumor necrosis factor and steroid metabolism in chronic heart failure: possible relation to muscle wasting. Journal of the American College of Cardiology 30: 997–1001. doi: 10.1016/s0735-1097(97)00262-3 9316530

[22] von Haehling S, Doehner W, Anker SD (2007) Nutrition, metabolism, and the complex pathophysiology of cachexia in chronic heart failure. Cardiovascular research 73: 298–309. doi: 10.1016/j.cardiores.2006.08.018 17034772

[23] Uchmanowicz I, Łoboz-Rudnicka M, Szeląg P, Jankowska-Polańska B, Łoboz-Grudzień K (2014) Frailty in heart failure. Current heart failure reports 11: 266–273. doi: 10.1007/s11897-014-0198-4 24733407

[24] Chaves PH, Varadhan R, Lipsitz LA, Stein PK, Windham BG, et al. (2008) Physiological complexity underlying heart rate dynamics and frailty status in community-dwelling older women. Journal of the American Geriatrics Society 56: 1698–1703. doi: 10.1111/j.1532-5415.2008.01858.x 19166446 PMC2848445

[25] Parvaneh S, Howe CL, Toosizadeh N, Honarvar B, Slepian MJ, et al. (2016) Regulation of cardiac autonomic nervous system control across frailty statuses: a systematic review. Gerontology 62: 3–15. 10.1159/000431285 PMC4930075 26159462

[26] Moghtadaei M, Jansen HJ, Mackasey M, Rafferty SA, Bogachev O, et al. (2016) The impacts of age and frailty on heart rate and sinoatrial node function. The Journal of physiology 594: 7105–7126. doi: 10.1113/JP272979 27598221 PMC5134407

[27] Afilalo J, Alexander KP, Mack MJ, Maurer MS, Green P, et al. (2014) Frailty assessment in the cardiovascular care of older adults. Journal of the American College of Cardiology 63: 747–762. doi: 10.1016/j.jacc.2013.09.070 24291279 PMC4571179

[28] Flint KM, Matlock DD, Lindenfeld J, Allen LA (2012) Frailty and the selection of patients for destination therapy left ventricular assist device. Circulation: Heart Failure 5: 286–293. doi: 10.1161/CIRCHEARTFAILURE.111.963215 22438521 PMC3869992

[29] Verma AK, Garg A, Xu D, Bruner M, Fazel-Rezai R, et al. (2017) Skeletal muscle pump drives control of cardiovascular and postural systems. Scientific reports 7: 1–8. doi: 10.1038/s41598-016-0028-x 28345674 PMC5366896

[30] Stehlik J, Schmalfuss C, Bozkurt B, Nativi-Nicolau J, Wegerich S, et al. (2018) Continuous wearable monitoring analytics predict heart failure decompensation: the LINK-HF multi-center study. Journal of the American College of Cardiology 71: A646-A646.

[31] Stehlik J, Schmalfuss C, Bozkurt B, Nativi-Nicolau J, Wohlfahrt P, et al. (2020) Continuous Wearable Monitoring Analytics Predict Heart Failure Hospitalization: The LINK-HF Multicenter Study. Circulation: Heart Failure 13: e006513. doi: 10.1161/CIRCHEARTFAILURE.119.006513 32093506

[32] Rens N, Gandhi N, Mak J, Paul J, Bent D, et al. (2021) Activity data from wearables as an indicator of functional capacity in patients with cardiovascular disease. Plos one 16: e0247834. doi: 10.1371/journal.pone.0247834 33760846 PMC7990307

[33] Association GAotWM (2014) World Medical Association Declaration of Helsinki: ethical principles for medical research involving human subjects. The Journal of the American College of Dentists 81: 14–18. 25951678

[34] Folstein MF, Folstein SE, McHugh PR (1975) “Mini-mental state”: a practical method for grading the cognitive state of patients for the clinician. Journal of psychiatric research 12: 189–198. doi: 10.1016/0022-3956(75)90026-6 1202204

[35] Nasreddine ZS, Phillips NA, Bédirian V, Charbonneau S, Whitehead V, et al. (2005) The Montreal Cognitive Assessment, MoCA: a brief screening tool for mild cognitive impairment. Journal of the American Geriatrics Society 53: 695–699. doi: 10.1111/j.1532-5415.2005.53221.x 15817019

[36] Charlson ME, Pompei P, Ales KL, MacKenzie CR (1987) A new method of classifying prognostic comorbidity in longitudinal studies: development and validation. Journal of chronic diseases 40: 373–383. doi: 10.1016/0021-9681(87)90171-8 3558716

[37] Kroenke K, Spitzer RL (2002) The PHQ-9: a new depression diagnostic and severity measure. SLACK Incorporated Thorofare, NJ. pp. 509–515.

[38] Panel on Prevention of Falls in Older Persons AGS, Society BG (2011) Summary of the updated American Geriatrics Society/British Geriatrics Society clinical practice guideline for prevention of falls in older persons. Journal of the American Geriatrics Society 59: 148–157. doi: 10.1111/j.1532-5415.2010.03234.x 21226685

[39] Rubenstein LZ, Vivrette R, Harker JO, Stevens JA, Kramer BJ (2011) Validating an evidence-based, self-rated fall risk questionnaire (FRQ) for older adults. Journal of Safety Research 42: 493–499. doi: 10.1016/j.jsr.2011.08.006 22152267

[40] Yardley L, Beyer N, Hauer K, Kempen G, Piot-Ziegler C, et al. (2005) Development and initial validation of the Falls Efficacy Scale-International (FES-I). Age and ageing 34: 614–619. doi: 10.1093/ageing/afi196 16267188

[41] Fieo RA, Mortensen EL, Rantanen T, Avlund K (2013) Improving a measure of mobility-related fatigue (the Mobility-Tiredness Scale) by establishing item intensity. Journal of the American Geriatrics Society 61: 429–433. doi: 10.1111/jgs.12122 23452001 PMC4047030

[42] Toosizadeh N, Mohler J, Lei H, Parvaneh S, Sherman S, et al. (2015) Motor performance assessment in Parkinson’s disease: association between objective in-clinic, objective in-home, and subjective/semi-objective measures. PloS one 10: e0124763. doi: 10.1371/journal.pone.0124763 25909898 PMC4409065

[43] Mohler MJ, Wendel CS, Taylor-Piliae RE, Toosizadeh N, Najafi B (2016) Motor performance and physical activity as predictors of prospective falls in community-dwelling older adults by frailty level: application of wearable technology. Gerontology 62: 654–664. doi: 10.1159/000445889 27160666 PMC5073011

[44] Aminian K, Najafi B, Büla C, Leyvraz P-F, Robert P (2002) Spatio-temporal parameters of gait measured by an ambulatory system using miniature gyroscopes. Journal of biomechanics 35: 689–699. doi: 10.1016/s0021-9290(02)00008-8 11955509

[45] Najafi B, Helbostad JL, Moe-Nilssen R, Zijlstra W, Aminian K (2009) Does walking strategy in older people change as a function of walking distance? Gait & posture 29: 261–266. doi: 10.1016/j.gaitpost.2008.09.002 18952435

[46] Jansen C-P, Toosizadeh N, Mohler MJ, Najafi B, Wendel C, et al. (2019) The association between motor capacity and mobility performance: frailty as a moderator. European Review of Aging and Physical Activity 16: 1–8. doi: 10.1186/s11556-018-0207-9 31624506 PMC6787993

[47] Hayes AF, Rockwood NJ (2017) Regression-based statistical mediation and moderation analysis in clinical research: Observations, recommendations, and implementation. Behaviour research and therapy 98: 39–57. doi: 10.1016/j.brat.2016.11.001 27865431

[48] Martínez-Ramírez A, Martinikorena I, Gómez M, Lecumberri P, Millor N, et al. (2015) Frailty assessment based on trunk kinematic parameters during walking. Journal of Neuroengineering and rehabilitation 12: 1–10. doi: 10.1186/1743-0003-12-1 26003560 PMC4443533

[49] Kressig RW, Gregor RJ, Oliver A, Waddell D, Smith W, et al. (2004) Temporal and spatial features of gait in older adults transitioning to frailty. Gait & posture 20: 30–35. doi: 10.1016/S0966-6362(03)00089-4 15196517

[50] Schwenk M, Mohler J, Wendel C, Fain M, Taylor-Piliae R, et al. (2015) Wearable sensor-based in-home assessment of gait, balance, and physical activity for discrimination of frailty status: baseline results of the Arizona frailty cohort study. Gerontology 61: 258–267. doi: 10.1159/000369095 25547185 PMC4452118

[51] Manini TM, Clark BC (2012) Dynapenia and aging: an update. Journals of Gerontology Series A: Biomedical Sciences and Medical Sciences 67: 28–40. doi: 10.1093/gerona/glr010 21444359 PMC3260480

[52] Evans W, Paolisso G, Abbatecola A, Corsonello A, Bustacchini S, et al. (2010) Frailty and muscle metabolism dysregulation in the elderly. Biogerontology 11: 527–536. doi: 10.1007/s10522-010-9297-0 20683658

[53] Roos MR, Rice CL, Vandervoort AA (1997) Age-related changes in motor unit function. Muscle & Nerve: Official Journal of the American Association of Electrodiagnostic Medicine 20: 679–690. doi: 10.1002/(sici)1097-4598(199706)20:6<679::aid-mus4>3.0.co;2-5 9149074

[54] Porter M, Vandervoort A, Lexell J (1995) Aging of human muscle: structure, function and adaptability. Scandinavian journal of medicine & science in sports 5: 129–142. doi: 10.1111/j.1600-0838.1995.tb00026.x 7552755

[55] Van Empel VP, Kaye DM, Borlaug BA (2014) Effects of healthy aging on the cardiopulmonary hemodynamic response to exercise. The American journal of cardiology 114: 131–135. doi: 10.1016/j.amjcard.2014.04.011 24852914

[56] Yataco AR, Fleisher LA, Katzel LI (1997) Heart rate variability and cardiovascular fitness in senior athletes. American Journal of Cardiology 80: 1389–1391. doi: 10.1016/s0002-9149(97)00697-8 9388127

[57] Romero-Ortuno R, Cogan L, O’Shea D, Lawlor BA, Kenny RA (2011) Orthostatic haemodynamics may be impaired in frailty. Age and ageing 40: 576–583. doi: 10.1093/ageing/afr076 21749997

[58] Bellumkonda L, Tyrrell D, Hummel SL, Goldstein DR (2017) Pathophysiology of heart failure and frailty: a common inflammatory origin? Aging cell 16: 444–450. doi: 10.1111/acel.12581 28266167 PMC5418206

[59] Weiss CO, Hoenig HH, Varadhan R, Simonsick EM, Fried LP (2010) Relationships of cardiac, pulmonary, and muscle reserves and frailty to exercise capacity in older women. Journals of Gerontology Series A: Biomedical Sciences and Medical Sciences 65: 287–294. 10.1093/gerona/glp147 PMC2822279 19822621

[60] Evrengul H, Tanriverdi H, Kose S, Amasyali B, Kilic A, et al. (2006) The relationship between heart rate recovery and heart rate variability in coronary artery disease. Annals of Noninvasive Electrocardiology 11: 154–162. doi: 10.1111/j.1542-474X.2006.00097.x 16630090 PMC7313315

