# Supplementary material for: The association between heart rate behavior and gait performance: The moderating effect of frailty
Source: PLoS One. 2022 Feb 16;17(2):e0264013. doi: 10.1371/journal.pone.0264013 (PMC8849485; doi:10.1371/journal.pone.0264013)
Supplement: S1 Table — Significant values are denoted by the asterisk symbol. (DOCX) [file pone.0264013.s002.docx]

| **HR Dynamics** | All Participants *r-*value (*p-*value) | | |
| --- | --- | --- | --- |
|  | Time to Peak HR | Increase Rate | Percent Increase |
| Stride Length | 0.4268 (<0.001*) | 0.1481 (0.1841) | 0.0561 (0.7429) |
| Stride Velocity | 0.4754 (<0.0001*) | 0.1549 (0.1648) | 0.0632 (0.5637) |
| Mean Swing Velocity | 0.5314 (<0.0001*) | 0.2786 (0.0113*) | 0.2000 (0.0663) |
| Double Support Duration | 0.5314 (<0.0001*) | 0.2786 (0.0113*) | 0.2001 (0.0663) |

Supplementary Table S1: Correlation analyses between baseline HR and HR dynamic parameters with gait parameters for all participants and for each frailty group. Significant values are denoted by the asterisk symbol.

| **HR Dynamics** | Non-frail *r-*value (*p-*value) | | |
| --- | --- | --- | --- |
|  | Time to Peak HR | Increase Rate | Percent Increase |
| Stride Length | 0.0322 (0.8758) | 0.0788 (0.7143) | 0.0100 (0.9613) |
| Stride Velocity | 0.1539 (0.4528) | 0.1058 (0.6226) | 0.0638 (0.7566) |
| Mean Swing Velocity | 0.0568 (0.7829) | 0.0677 (0.7530) | 0.1192 (0.5620) |
| Double Support Duration | 0.0569 (0.7824) | 0.0679 (0.7526) | 0.1192 (0.5616) |

| **HR Dynamics** | Pre-frail/Frail *r-*value (*p-*value) | | |
| --- | --- | --- | --- |
|  | Time to Peak HR | Increase Rate | Percent Increase |
| Stride Length | 0.5079 (<0.001*) | 0.0080 (0.9527) | 0.2054 (0.1185) |
| Stride Velocity | 0.5733 (<0.0001*) | 0.1534 (0.2502) | 0.0475 (0.7204) |
| Mean Swing Velocity | 0.5868 (<0.0001*) | 0.2908 (0.0268*) | 0.1175 (0.3752) |
| Double Support Duration | 0.5868 (<0.0001*) | 0.2908 (0.0268*) | 0.1175 (0.3755) |

| **Baseline HR** | All Participants *r-*value (*p-*value) | | |
| --- | --- | --- | --- |
|  | Time to Peak HR | Increase Rate | Percent Increase |
| Stride Length | 0.1101 (0.3188) | 0.0663 (0.5488) | 0.0960 (0.3850) |
| Stride Velocity | 0.0470 (0.6709) | 0.0652 (0.5556) | 0.1050 (0.3417) |
| Mean Swing Velocity | 0.0379 (0.7324) | 0.0133 (0.9042) | 0.0060 (0.9568) |
| Double Support Duration | 0.0380 (0.7314) | 0.0138 (0.9038) | 0.0001 (0.9564) |

| **Baseline HR** | Non-frail *r-*value (*p-*value) | | |
| --- | --- | --- | --- |
|  | Time to Peak HR | Increase Rate | Percent Increase |
| Stride Length | 0.0300 (0.8842) | 0.0581 (0.7781) | 0.0928 (0.6520) |
| Stride Velocity | 0.0140 (0.9457) | 0.0614 (0.7655) | 0.1034 (0.6153) |
| Mean Swing Velocity | 0.0001 (0.9818) | 0.0316 (0.8783) | 0.0102 (0.9604) |
| Double Support Duration | 0.0001 (0.9813) | 0.0317 (0.8777) | 0.0101 (0.9609) |

| **Baseline HR** | Pre-frail/Frail *r-*value (*p-*value) | | |
| --- | --- | --- | --- |
|  | Time to Peak HR | Increase Rate | Percent Increase |
| Stride Length | 0.0931 (0.4872) | 0.1703 (0.2010) | 0.1892 (0.1546) |
| Stride Velocity | 0.0106 (0.9369) | 0.1587 (0.2342) | 0.1922 (0.1483) |
| Mean Swing Velocity | 0.0001 (0.9573) | 0.0726 (0.5881) | 0.0540 (0.6872) |
| Double Support Duration | 0.0070 (0.9584) | 0.0726 (0.5882) | 0.0539 (0.6875) |
